# Implementation of a trauma-informed care initiative at FQHCs in Texas

**DOI:** 10.3389/frhs.2026.1881638

**Published:** 2026-08-12

**Authors:** Rachel Barnes, Jihye Choi, Qiheng Yan, Aimee Rachel, Efrat K. Gabay, Paula M. Cuccaro

**Affiliations:** 1 Texas Epidemic Public Health Institute, The University of Texas Health Science Center at Houston School of Public Health, Houston, TX, United States; 2 Department of Health Disparities Research, The University of Texas MD Anderson Cancer Center, Houston, TX, United States; 3 Department of Health Promotion and Behavioral Sciences, The University of Texas Health Science Center at Houston School of Public Health, Houston, TX, United States; 4 Trauma Informed Restoration Group, PLCC, Denton, TX, United States; 5 Center for Health Promotion and Prevention Research, The University of Texas Health Science Center at Houston School of Public Health, Houston, TX, United States

**Keywords:** community health centers, implementation, training, trauma, trauma-informed care

## Abstract

**Introduction:**

Trauma-informed care recognizes the high prevalence and impact of trauma across the healthcare continuum, and its implementation across healthcare settings has varied. We describe the development and implementation of a trauma-informed care initiative at community health centers across Texas.

**Methods:**

Using an inquiry-based learning approach, a train-the-trainer model disseminated trauma-informed principles across participating health centers. This approach was evaluated using formative, summative, and organizational assessments as well as qualitative interviews with trained staff.

**Results:**

34 health centers participated in the trauma-informed care initiative. We observed significant improvements in organizational assessment subdomains and an average score of 73.9% in summative assessments. Trained staff shared difficulties related to learner engagement, time, leadership buy-in, and self-efficacy to train others, however, successes included enhanced cross-departmental collaboration and incorporation of trauma-informed principles in everyday practice.

**Discussion:**

Trauma-informed care has the potential to transform healthcare delivery to ensure staff, patient, and organizational wellbeing. We describe a novel approach to implementing trauma-informed care at health centers that shows promise for organizational transformation. Future programs should consider pre-post knowledge assessments, leadership engagement, and sustainability methods such as certifications or continuing education.

## 1 Introduction

Trauma is any distressing event that results in intense fear, dissociation, or other disruptive feelings with enduring negative effects on a person's functioning (1). Trauma has become an increasingly alarming concern in the 21st century (2). A prominent source of trauma is adverse childhood experiences, or ACEs, such as abuse, violence, or neglect that occur between the ages of 0 and 17 years (3). More than 75% of high school students in the United States have experienced at least one ACE, and nearly 20% have experienced four or more ACEs (4). Additionally, nearly 90% of US adults have experienced a traumatic event, and multiple exposures are common (5). The landmark Felitti et al. ACEs study in 1998 highlighted the strong relationship between trauma and chronic health conditions such as heart disease and cancer. Trauma is also linked to mental health conditions such as depression, PTSD, and substance use (6). Moreover, trauma is associated with increased healthcare utilization and prescription medications as well as decreased quality of life (6).

Federally qualified health centers (FQHCs) are safety-net providers that offer comprehensive healthcare to any patient, including historically underserved populations as well as the uninsured/underinsured. More than 90% of FQHC patients have experienced a traumatic event, and nearly 80% have experienced multiple traumatic events (7). At the same time, FQHC staff are at an elevated risk of secondary or vicarious trauma when serving patients who have gone through traumatic experiences (8). The COVID-19 pandemic only worsened burnout and trauma among these frontline workers (9). Vicarious trauma can threaten staff performance and wellbeing, contributing to greater turnover, shortages, errors, and care disruptions (10, 11).

The effects of trauma on patients and staff highlight the need for a trauma-informed approach to care at FQHCs. Trauma-informed care (TIC) recognizes the high prevalence and impact of trauma across the continuum of care. Hopper et al. defines TIC as a healthcare delivery approach “that is grounded in an understanding of and responsiveness to the impact of trauma, that emphasizes physical, psychological, and emotional safety for providers and survivors, and that creates opportunities for survivors to rebuild a sense of control and empowerment” (12). A trauma-informed approach can prevent re-traumatization, build patient trust, and reduce the long-lasting effects of trauma (13–15). At the staff level, TIC has been shown to foster a culture of staff wellness by strengthening relationships with management, improving staff self-efficacy, and increasing staff commitment (16). Additionally, TIC can reduce staff turnover, use of sick time, and liability-related expenses (17, 18). Despite the need for trauma-informed organizational transformation at FQHCs, few programs have implemented such programs in health centers. This study evaluates the implementation of a TIC initiative at FQHCs across Texas from 2019 to 2024. This study was reviewed by the University of Texas Health Science Center Houston Committee for the Protection of Human Subjects and deemed to be quality improvement research.

## 2 Materials and methods

**Figure 1:**
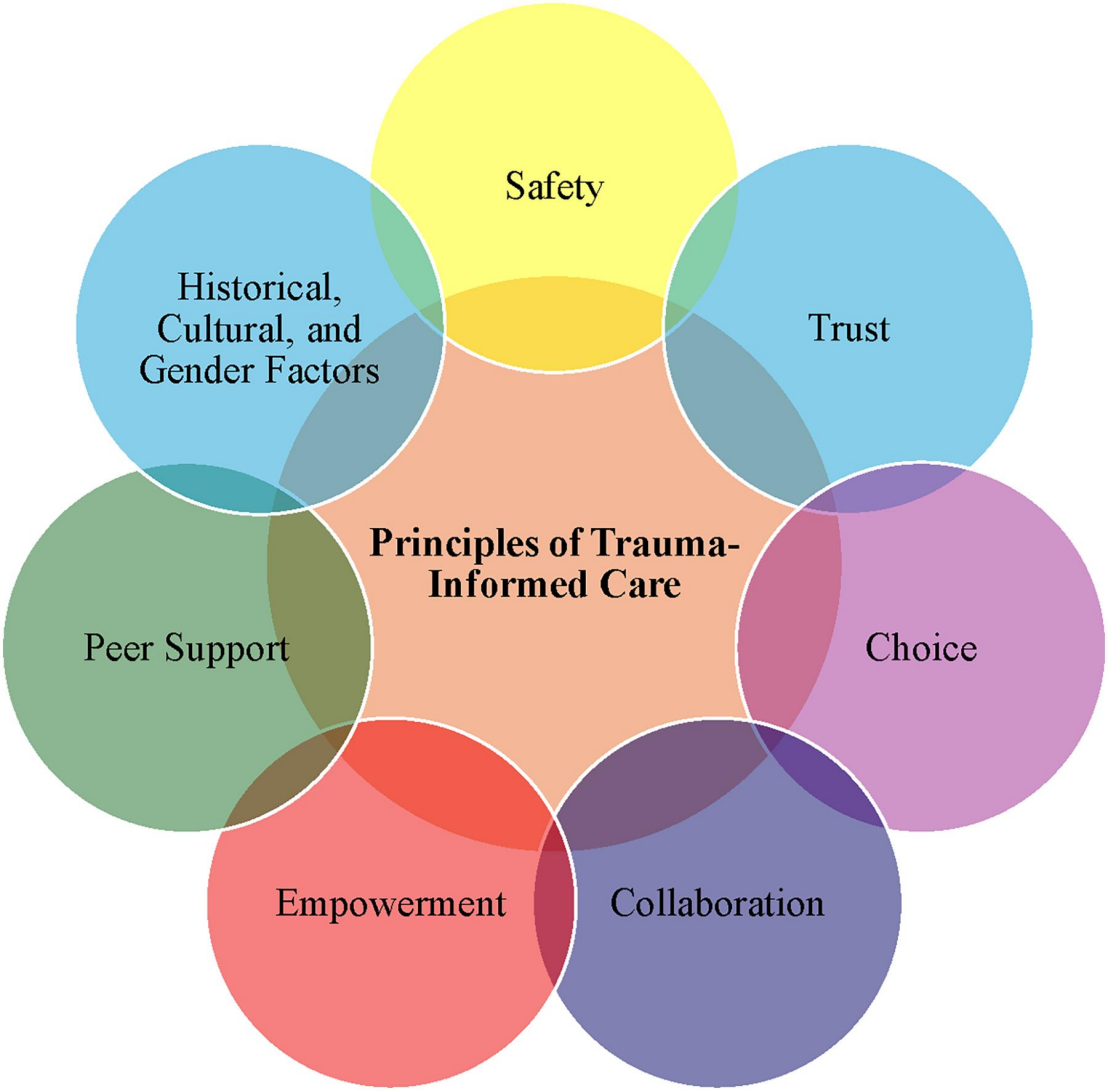
Seven principles of TIC, adapted from SAMHSA (23).

**Figure 2:**
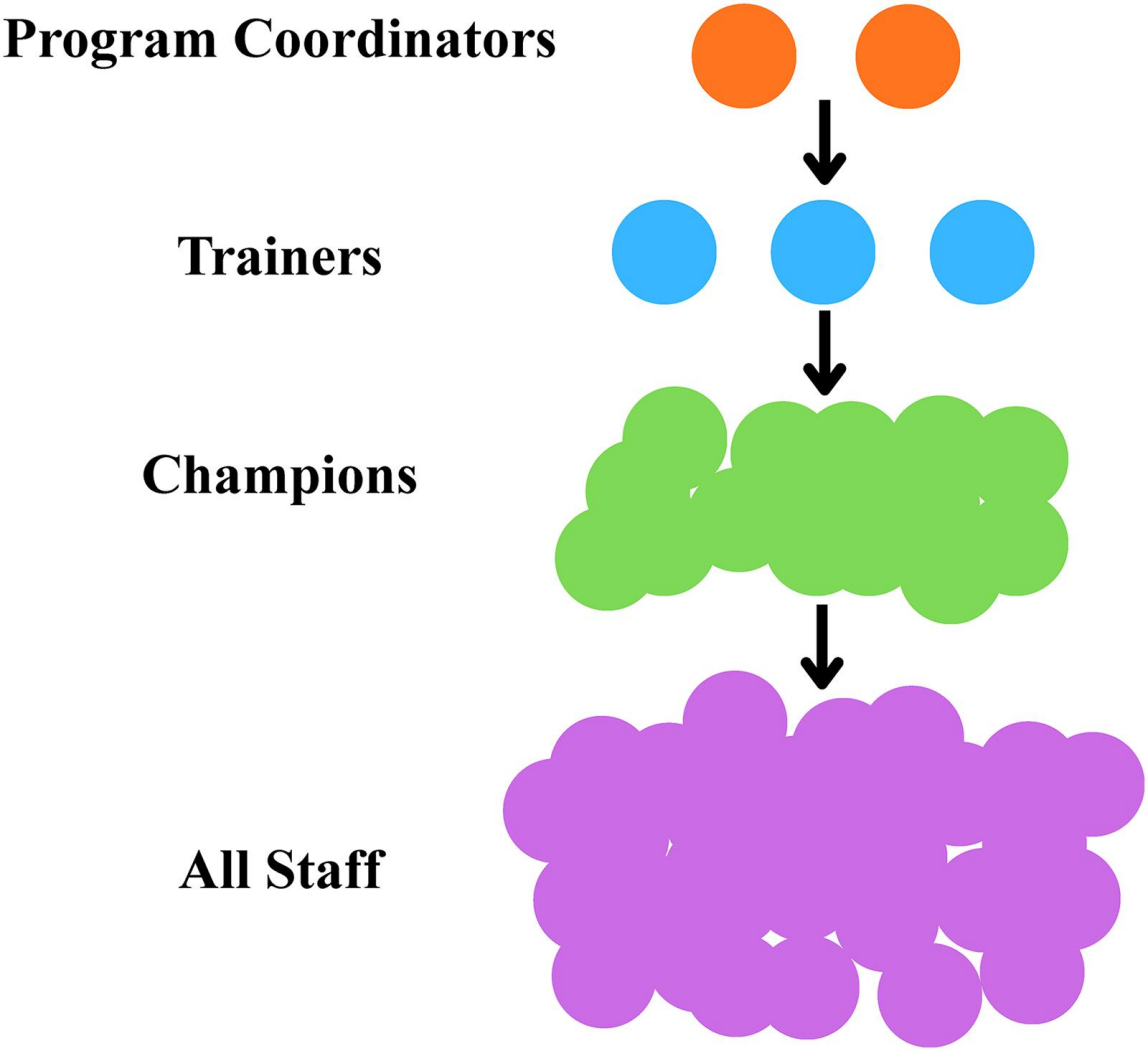
Illustration of the train-the-trainer model to disseminate TIC throughout participating health center staff.

In April 2019, a primary care association (PCA) based in Texas launched a TIC program across multiple FQHCs and look-alike health centers in the state. The program aimed to shift the culture of participating health centers to one that provided direct patient care in a trauma-informed environment. Specifically, the end goals of the TIC program were 1) establish trauma-informed care as a critical component of health centers; 2) ensure physical, psychological, cultural, moral, emotional, and spiritual safety; 3) enhance trustworthiness and transparency in policies and interactions; 4) develop, sustain, and empower a trauma-informed workforce; 5) integrate systems of care that establish and support collaboration; and 6) acknowledge historical, cultural, and gender factors to provide choice. These principles aim to reduce re-traumatization and promote healing and resilience among all individuals in health centers. Health centers were recruited through in-person interactions and word-of-mouth at PCA meetings, conferences, and other gatherings, leveraging the TIC coordinators' established relationships. A group of *Trainers* from consecutive cohorts of health centers participated in a one-year intensive training provided by two program coordinators between 2019 and 2024 that equipped them to practice TIC principles (see Figure 1). Following the training, in line with a train-the-trainer model of dissemination, Trainers trained *Champions* that represented each department across the health centers. Finally, Champions disseminated TIC throughout the health center with all remaining staff through *Departmental* trainings. Figure 2 demonstrates how TIC spread throughout the health centers for organizational transformation. Once Departmental trainings were completed, the health center had completed TIC training and focus shifted to integration of TIC principles into everyday practice.

### 2.1 TIC trainer curriculum

The TIC Trainer curriculum is centered around a central truth: the implementation of TIC can cause change. Using an inquiry-based learning (IBL) approach, the curriculum emphasizes questions and problems to contextualize the content in a learner-centered manner (19). The curriculum additionally encourages a community of learners to enhance engagement through social interaction (20). Through both inductive and deductive reasoning, learners formulate and test hypotheses, engage in observation and reflection, and critically consider the perspectives and ideas of others (19) This strategy places questioning, exploration, and shared meaning-making at the core of the learning experience, in line with Knowles' adult learning principles which emphasize applicable knowledge (19, 21). IBL has been shown to improve critical thinking skills, motivation, and knowledge, which are all essential to maximize Trainer commitment and training of subsequent Champions and staff (22). The content included in the curriculum was adapted from the Substance Abuse and Mental Health Administration (SAMHSA)'s TIC framework core principles, highlighted in Figure 1, as well as their practical guide and treatment improvement protocol (23–25). Additional topics included change and self-care to support health center and staff needs as the organization goes through transformation.

The TIC curriculum had four components: written, taught, assessed, and reflective curriculum. The written curriculum provided a framework for the knowledge to be gained with each of the 10 units of study, keeping in mind Trainers' prior knowledge. The taught curriculum encompassed what is instructed to the Trainers, including prior knowledge, multimedia presentations, interactive discussions, interviews, literature, and collaboration. The assessed curriculum included formative and summative assessments to evaluate Trainer knowledge during and at the conclusion of training. Finally, the reflective curriculum provides notated information to the TIC coordinators to reflect on the Trainer experience and make changes as needed to enhance learning and engagement.

The structure of the training was multilayered and consisted of three main components: 1) in person “foundational series” training sessions, 2) virtual learning community sessions, and 3) virtual coaching calls. The foundational training consisted of four 8-hour in-person sessions. During the in-person training sessions, all participants gathered to learn about TIC foundations and other essential topics related to TIC. Guest content experts provided additional knowledge sharing. The virtual learning community sessions were held monthly for 1.5 hours and focused on a different TIC concept in each session. Questions or hands-on activities were designed to promote curiosity about the upcoming learning community sessions. Finally, 1-hour tailored, discussion-based coaching calls were virtually provided by the program coordinators every month to each site-based team of Trainers. These calls offered space to examine concepts in greater depth, address challenges, and support training to ensure their understanding of TIC and its application in their practice. After 12 months, the coaching calls transitioned to implementation-focused calls to provide continued technical assistance. The training overall encouraged Trainers to reflect on the learned concepts and collaborate with fellow TIC Trainers as they moved through the TIC transformation. The structure and content of the TIC Trainer training are summarized in Table 1.

**Table 1:** One-year TIC trainer training structure and learning topics.

Month	Component	Topic
1	Foundational Series	TIC foundations and kick-off
2	Learning community session	Change and self-care
	Coaching call	
3	Learning community session	Governance, leadership, and policy
	Coaching call	
4	Learning community session	Physical environment and progress monitoring
	Coaching call	
5	Foundational series	Justice, equity, diversity, and inclusion
	Learning community session	Cross-sector collaboration and engagement
	Coaching call	
6	Learning community session	How to train
	Coaching call	
7	Learning community session	Workforce development
	Coaching call	
8	Learning community session	Screening and assessing
	Coaching call	
9	Foundational series	Brain development and non-medical drivers of health
	Learning community session	TIC reflective supervision
	Coaching call	
10	Learning community session	Resilience and transformation
	Coaching call	
11	Learning community session	TIC peer panel discussion
	Coaching call	
12	Foundational series	Sustain and spread
	Learning community session	Sustainability
	Coaching call	
13+	Coaching calls	Implementation

Critical to the curriculum were formative and summative assessments to assess Trainer knowledge and the overall program. Formative assessments inform and tailor student learning, whereas summative assessments inform program effectiveness (26). The formative assessments functioned as “homework” to be completed between learning community sessions. Each formative assessment aligned with the TIC principle to be taught during a foundational or learning community session. This ongoing process enabled instructors and staff to monitor trainees' learning progress throughout instruction. The primary goal was to identify misconceptions, challenges, and learning gaps, allowing timely intervention and support. This approach emphasized growth and skill development rather than performance appraisal through graded assignments. By engaging in formative assessments, Trainers took ownership of their learning, recognizing that the focus was on enhancing skills and knowledge. Formative assessment methods included self-assessment, peer feedback, and instructor evaluation through tools such as writing assignments, practical group exercises, and discussions. The use of formative assessments also supported tailored instructional strategies, fostering a more personalized learning experience.

Upon completion of the TIC learning community sessions, Trainers undertook a final summative assessment to evaluate learner comprehension and the effectiveness of content delivery. This assessment measured learning outcomes, proficiency, and progress toward TIC transformation against a standardized rubric. The TIC summative assessment was designed to provide a comprehensive overview of trainees' knowledge while guiding TIC coordinating staff to reflect on instructional fidelity. Importantly, this summative tool was aligned with the formative assessments used throughout the learning process by evaluating the same topics with the same language, ensuring continuity and coherence in evaluating trainee growth. The summative assessment consisted of 42 multiple choice, open-ended, and Likert scale questions that evaluated both TIC knowledge and implementation at participating health centers. The multiple choice questions focused on knowledge with specific questions aligned with each TIC principle and were scored either correct or incorrect. The open-ended and Likert scale questions provided a picture of perceived TIC implementation at the participating health center; rather than correct or incorrect answers, these questions illustrated implementation fidelity to inform the focus of ongoing implementation calls between Trainers and TIC coordinators.

### 2.2 Selection of trainers

For this intervention, each participating health center assembled a training team comprising three Trainers representing different professional roles: a medical provider (e.g., physician or dentist), a behavioral health specialist, and a third staff member who could be either clinical or non-clinical (e.g., an administrator). Health center leadership identified staff with both available time and interest in the program. The composition of the Trainer team was intentional; by integrating diverse roles, the team could distribute responsibilities more evenly and reduce the risk of overburdening any single training team member as well as account for possible turnover. Each role also contributed essential expertise. A medical provider was needed given that trauma frequently manifests in examination rooms, and clinical insight is critical for recognizing and responding to it in real time. A behavioral health specialist was included because trauma is fundamentally connected to mental and behavioral health, and patients identified with trauma-related needs often require specialized follow-up or referral. As for the third member, health centers were given flexibility in selecting someone who could help sustain the training process, especially given the heavy clinical demands on medical and behavioral staff. Bringing these three roles together also acknowledged the different training cultures across medical, behavioral, and non-clinical teams and helped reduce the misconception that trauma is solely the domain of behavioral health departments.

### 2.3 Training champions

Once Trainers completed the TIC training curriculum, they designated program Champions and delivered a condensed version of the same training to them, following the train-the-trainer model (Figure 1). The Champions are identified by the Trainers as representatives of each department within their health center; Trainers worked with leadership to identify staff with available time and interest in participating. The Trainers passed along the condensed TIC curriculum and prepared Champions to train colleagues and advocate for TIC within their respective departments. This cascading structure enabled broad organizational reach and sustainability, with a ripple effect shown in prior studies to facilitate system-wide transformation and reinforce cultural change (27). By equipping Champions across all areas, the program fostered a culture of shared responsibility for trauma-informed practices throughout the organization.

### 2.4 Evaluation

In addition to the Trainer formative and summative assessments, all employees at participating sites anonymously completed a comprehensive Organizational Assessment (OA) at baseline, 18 months, and annually thereafter. The OA was derived from the Trauma-Informed Organizational Toolkit and used a 5-point Likert scale, where 5 indicated “strongly agree” (the health center incorporates the TIC practice) and 1 indicated “strongly disagree” (the health center does not incorporate the TIC practice) (28). The OA includes five domains of interest: Supporting Staff Development, Creating a Safe and Supportive Environment, Assessing and Planning Services, Involving Patients, and Adapting Policies. Each domain encompasses subdomains that more specifically ask about TIC practices. Survey responses were aggregated to understand overall TIC implementation. This assessment was critical for evaluating the impact of the TIC training intervention and guiding implementation plans. The OA measured organizational cultural shifts and the extent to which TIC had been integrated. Honest staff responses highlighted areas for improvement in programs and the work environment, informed professional development planning, and guided organizational policy changes. The results supported the TIC transformational process by providing actionable data to tailor and strengthen site-specific implementation strategies.

To analyze the effectiveness of the TIC initiative on organizational transformation, we conducted a longitudinal observational design. Data were collected at five key timepoints across multiple cohorts of participating health centers as previously described, with organizational assessments serving as the data source. We employed linear mixed-effects models to evaluate changes in TIC-related practices over time, incorporating time-varying treatment effects to capture changes over time. Timepoints were modeled as fixed effects, and responses were clustered by implementation cohort by including cohort as a random intercept. Because the OA survey was anonymous and individual staff responses were not linked across time, clustering was not modeled at the respondent level. We conducted model diagnostics, including residual plots, normality checks, and Cook's distance, to confirm the appropriateness of the models. We performed subdomain-level analyses to assess improvements across various domains of TIC practices, such as staff development, supportive environments, and trauma-specific interventions. These analyses enabled a comprehensive evaluation of the TIC initiative's impact on organizational transformation, with a focus on identifying statistically significant changes between baseline and subsequent follow-up assessments. This is an interim analysis evaluating data collected through June 2024, and subsequent assessments will further evaluate the sustained impact of the TIC initiative as additional data become available.

We also conducted qualitative evaluations to gain insight into Trainers' and Champions' perceptions of TIC implementation using semi-structured interviews. The research team developed a semi-structured interview guide consisting of core questions and optional probes. Interview topics explored participants' overall perceptions of the program, successes and challenges encountered during training, and recommendations for additional resources or activities that could strengthen the training program. Potential participants were identified through the TIC program coordinators. The research team emailed invitation letters to all Trainers and Champions, and those who expressed interest were scheduled for an individual interview. All interviews were conducted remotely via Zoom, and verbal informed consent was obtained from each participant before the interview began. Interviews lasted approximately 30 min on average, and participants received a $25 electronic gift card upon completion. All interviews were conducted by a research coordinator trained in qualitative research methods. In tandem with data collection, the research team met weekly to debrief emerging findings, clarify uncertainties, refine the interview guide as needed, and promote consistency and completeness in the qualitative data collection process.

Data were analyzed concurrently with data collection using Rapid Assessment Procedures (RAP), an approach well suited for implementation research and dynamic healthcare settings where timely evaluation is needed (29). RAP facilitates the generation of actionable findings while maintaining methodological rigor, which is particularly useful for informing intervention refinement and implementation (30). Each interview transcript was summarized using a standardized template organized by interview topic. These summaries were then synthesized into matrices that enabled comparisons across participants for each interview question and supported the identification of recurring themes. Salient quotations were extracted to illustrate key findings.

## 3 Results

**Figure 3:**
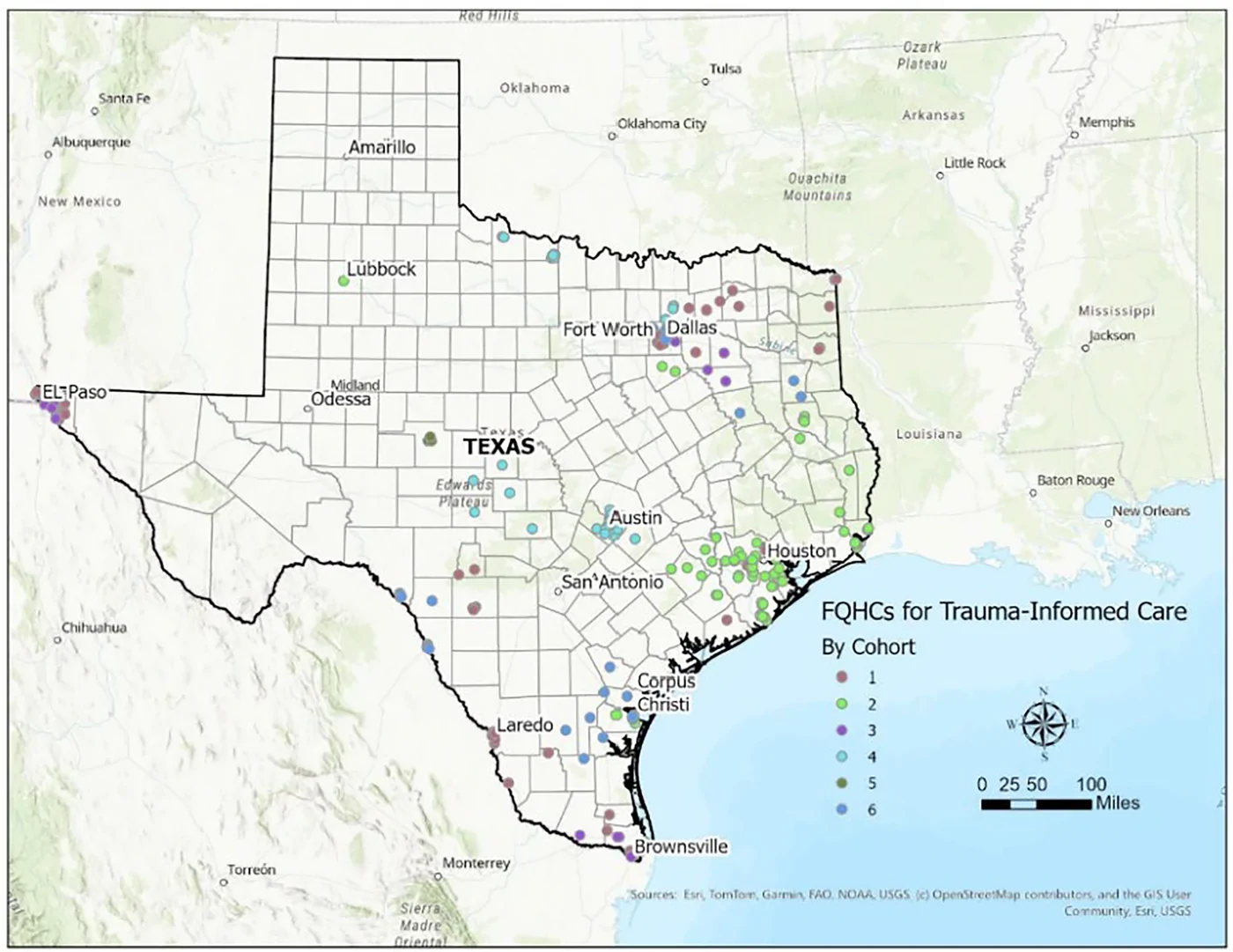
Map of health center sites participating in TIC across Texas divided by cohort.

Over the course of the TIC initiative, a total of 34 health centers and 120 Trainers participated across 240 sites. Due to staff turnover and health centers withdrawing from a cohort and rejoining in a later cohort, some health centers had more than three Trainers participate in TIC training. Of the participating health centers, 32 are designated FQHCs, one is a look-alike, and one is a Ryan White HIV/AIDS funded medical provider. Figure 3 shows the distribution of health center sites (including all clinic sites within a single FQHC system) across Texas and the six cohorts. The health centers were distributed across urban (61.8%), rural (35.3%), and frontier (2.9%) regions of the state.

### 3.1 Assessments

**Figure 4:**
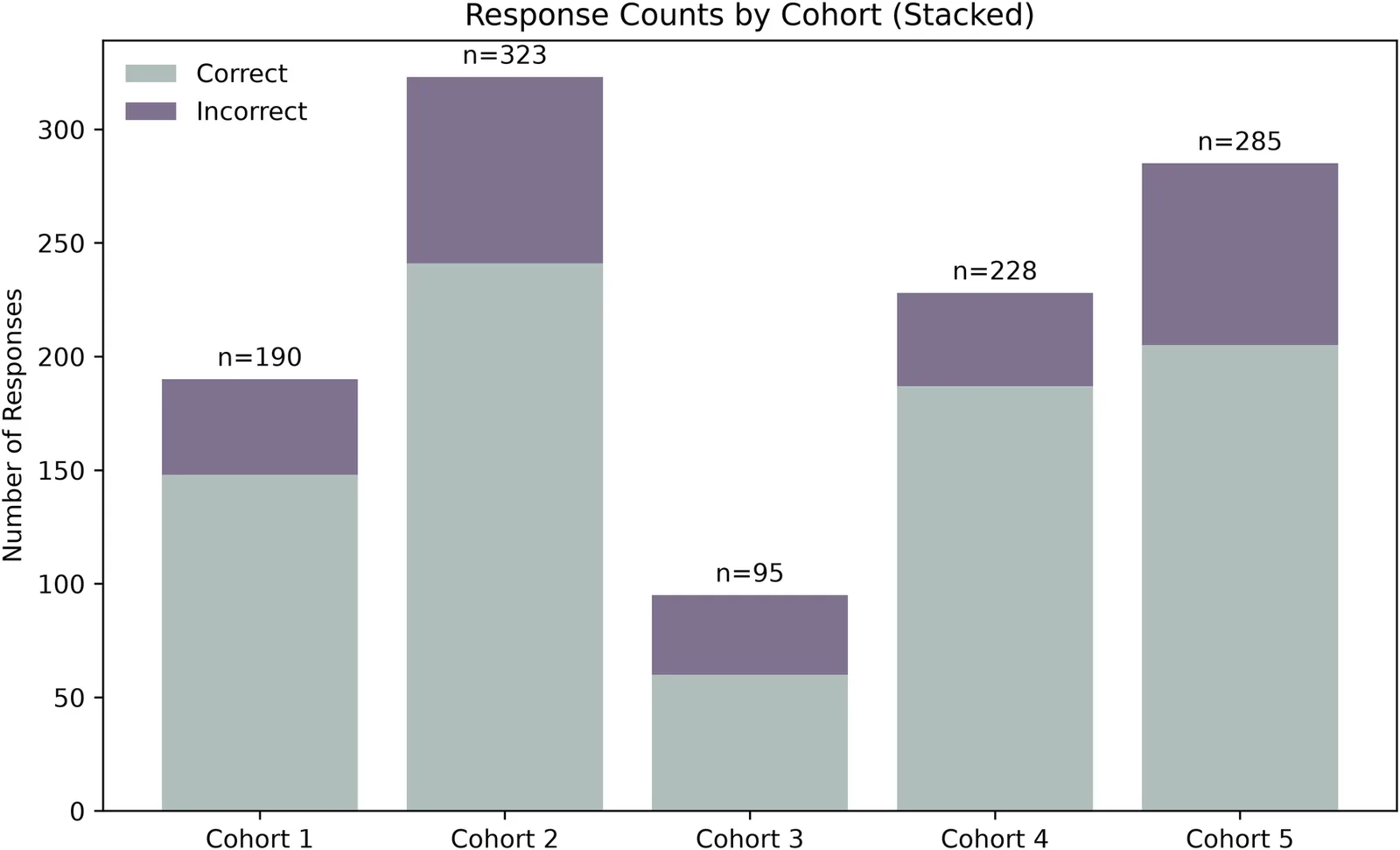
Summative assessment responses by cohort.

The summative assessment received 1,121 responses across the 5 cohorts that completed the TIC training. Implementation of TIC ended before cohort 6 was able to complete the summative assessment. Figure 4 shows the number of correct responses across the cohorts with an average of 73.9% correct.

**Figure 5:**
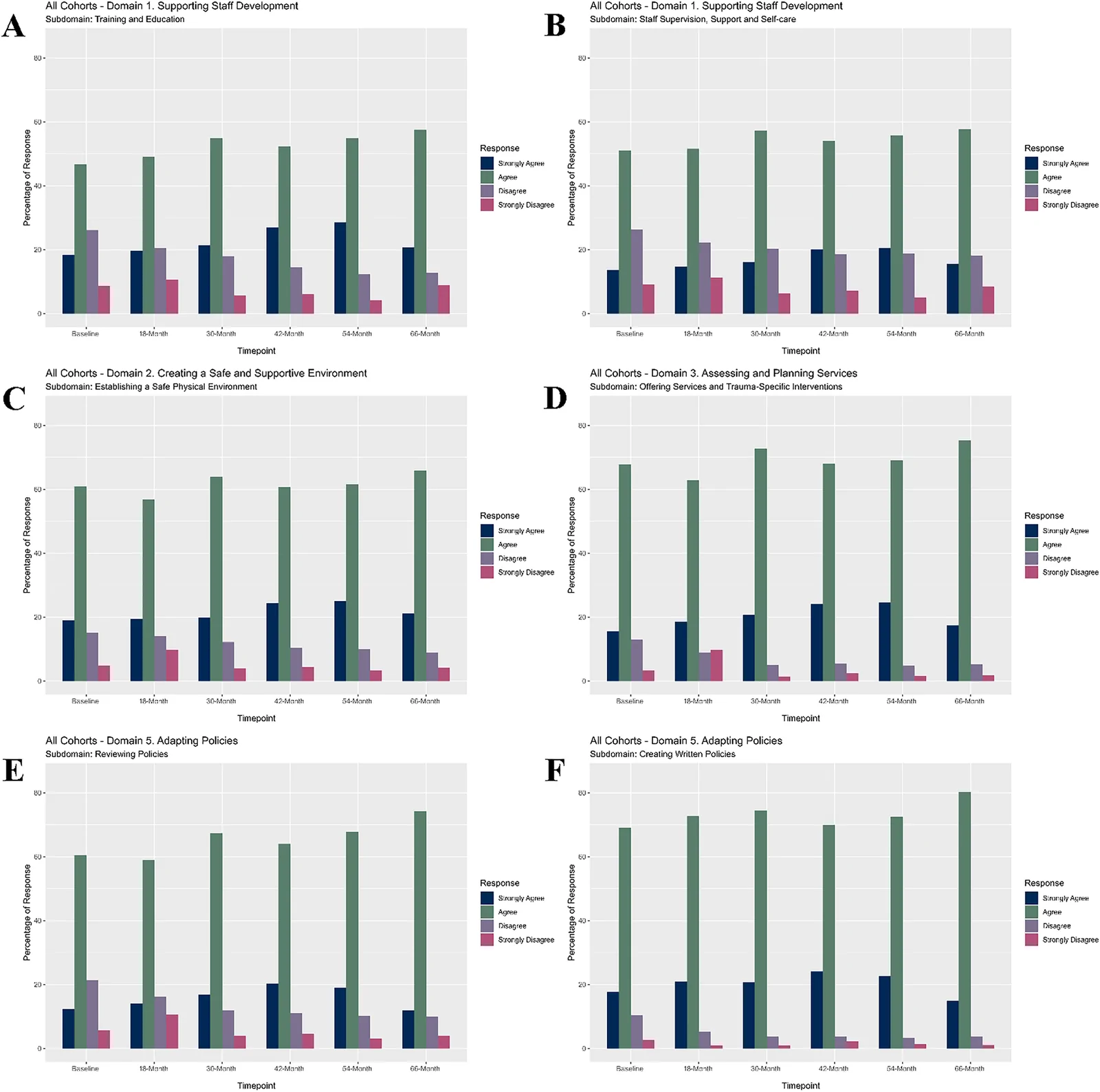
Aggregated organizational assessment responses from baseline to 66 months from the subdomains **(A)** training and education, **(B)** staff supervision, support, and self-care, **(C)** establishing a safe physical environment, **(D)** offering services and training-specific interventions, **(E)** reviewing policies, and **(F)** creating written policies.

The organizational assessment included 8,077 staff survey responses from five cohorts across five time points. Cohort 6 was excluded from analysis as implementation of TIC ended prior to the cohort being able to complete the OA. Figure 5 illustrates the distribution of responses across cohorts and time. A subdomain analysis examined changes from baseline to the latest available follow-up time point for each cohort, defined as 54 months for Cohorts 1 and 2, 30 months for Cohort 3, and 18 months for Cohort 4 and 5. The largest statistically significant improvement was observed for Training and Education (*β* = 0.3136, SE = 0.0101, *p* < 0.001), followed by Offering Services and Trauma-Specific Interventions (*β* = 0.1727, SE = 0.0187, *p* < 0.001), Staff Supervision, Support, and Self-Care (*β* = 0.1638, SE = 0.0117, *p* < 0.001), and Reviewing Policies (*β* = 0.1611, SE = 0.0213, *p* < 0.001). Significant improvements were also observed for Establishing a Safe Physical Environment (*β* = 0.1111, SE = 0.0122, *p* < 0.001), Creating Written Policies (*β* = 0.1084, SE = 0.0134, *p* < 0.001), Conducting Intake Assessments (*β* = 0.0899, SE = 0.0070, *p* < 0.001), Establishing a Supportive Environment (*β* = 0.0800, SE = 0.0069, *p* < 0.001), and Developing Goals and Plans (*β* = 0.0550, SE = 0.0172, *p* = 0.001). Involving Current and Former Patients was the only subdomain without a statistically significant change (*β* = 0.0132, SE = 0.0191, *p* = 0.49). Figure 5 illustrates aggregated responses for selected subdomains over the course of the data collection period.

### 3.2 Qualitative interviews

We interviewed TIC Trainers and Champions (*n* = 90) across the six cohorts annually to better understand their perspectives on TIC training and implementation. Their responses provided enhanced insights into the curriculum and implementation strategies as well as overall organizational transformation.

Trainers shared that they appreciated the organization and structure of the TIC curriculum as well as the expertise and availability of the coordinators throughout the training. One Trainer shared, “From an instructor standpoint, I think their training is wonderful, it's very well-organized and informative, and it's a safe environment.” (Trainer 9, Year 4). Another shared, the TIC coordinators “made themselves available if the team members were unclear or had questions” (Trainer 7, Year 3). Trainers and Champions additionally shared that the video, roleplaying, and mindfulness (e.g., music and art) components of the curriculum were particularly helpful in recognizing and applying TIC principles. For example, “I really liked when [they] gave an actual scenario because, at first, I really wasn't sure what trauma-informed was until [they] gave a scenario like, ‘Well, if this happened, how would you respond?” (Champion 1, Year 1).

There were, however, suggestions for improvement of the program as well. Over the years, both Trainers and Champions noted that the content could be lengthy, complex, and abstract. Trainers expressed frustration with the length of the formative assessments, with one reporting that they “completely discarded the homework assignments” (Trainer 12, Year 5). Participants also asked for more specific examples and trainings tailored to job types since the content could be focused on clinical or upper management roles at times. A Trainer suggested, “It will be important to improve and tailor the curriculum to our needs and make every opportunity with the training to make it super relevant to people who feel this” (Trainer 10, Year 4). Early feedback suggested including cultural and racial/ethnic sensitivity in the trainings because “just knowing a little bit more or understanding those perspectives would be helpful” (Champion 3, Year 3).

While engagement in TIC was noted as high during years 1 and 2 of implementation, a decline in learner engagement was seen with time. In Year 1, Trainers shared, “We received positive response[s] and people wanted to continue it because they saw the value in it,” and “We did have a good response from our staff when we met with them to talk about it” (Trainer 1, Year 1; Trainer 2, Year 1). By Year 5, a Trainer explained:

Here's a PowerPoint, and it has 60 slides, right? And I usually tell people, and probably the average person reads one and two. And by the tenth one, most people just click on it so they can get to the end, and they were not even reading, right? They were just, like, clicking next, next, next, because ‘I’ want to be done. So, I think that that's the part that's a little bit challenging sometimes. Making that training engaging. (Trainer 13, Year 5)

The COVID-19 pandemic presented additional challenges in terms of clinical priorities and shifting to hybrid or virtual learning modalities. A temporary pause to TIC training was followed by a shift to virtual platforms. Trainers felt that engagement diminished during this period, especially among Champions, due to distance and staff turnover. A Trainer shared, “We had to focus all of our efforts [on] the COVID situation, how to arrange the operations. So, some meetings and trainings have been placed on hold” (Trainer 3, Year 2). Another Trainer explained:

They [Champions] could be stronger as a team if they had been in the same room sharing the same story and looking at the same things all at the same time. As for me, working from home made it more difficult for us to choose our Champions so it slowed down our progress (Trainer 4, Year 2).

Beyond the COVID-19 pandemic, lack of time, funding, and leadership buy-in made prioritizing TIC training and implementation difficult across cohorts. Trainers were often also clinicians that had to consider balancing their time with patients and with training Champions. By Year 5, a Trainer shared:

Back when I was involved, I do feel like we had buy-in from leadership. However, it didn't seem feasible anymore. Priorities seemed different so we took a different approach…I think lack of support …from administration to actually make that happen. We can schedule, we can coordinate things with [agency] to do the meetings, but if there's no commitment to actually allow the staff one hour to do the trainings, then it's not going to happen (Trainer 14, Year 5).

The Trainers also had limited self-efficacy in training Champions throughout their health centers as part of the train-the-trainer model. A Trainer shared that “They gave us all the knowledge that we need, but I’m not 100% sure how I can train our Champions” (Trainer 8, Year 3). The large volume of materials handed out each session could be “overwhelming” for Trainers to prioritize what Champions needed to be trained on during their limited time together. Another Trainer noted about their experience training Champions, “Some of it is that people may have been a bit intimidated just because it is a lot of curriculum” (Trainer 5, Year 2).

Nevertheless, the Trainers and Champions saw transformation in their health centers as TIC was disseminated. They shared that the added TIC principle on self-care (e.g., using sick days for mental health) was readily applied, especially due to “collective trauma [from] COVID… providers and health workers all need that support, too” (Champion 2, Year 2). Staff across the clinics were actively thinking about TIC and how it could be integrated among themselves and with patients. Increased interdepartmental communications broke down barriers between staff, allowing for shared understanding of work experiences. A specific example of TIC implementation illustrates impacts on patient care:

Before some staff were taking a lot of these personally saying that patients were being rude. Now you start hearing them talking, “Well, what do you think they might have been going through? Or what do you think was happening?’ And then it disseminates into everything like when we put signs like no cell phones in the rooms. The behaviorist would bring it up and say, “Wait a minute. Remember trauma-informed care, we’re supposed to go positive, not no.’ There are conversations about it, which makes me happy. People are really thinking about TIC (Trainer 11, Year 4).

Finally, Trainers and Champions also provided ideas for sustainability. One Champion suggested continuing education units centered around TIC for clinicians, while several Trainers and Champions encouraged the incorporation of TIC into employee onboarding. “As staff [are] coming in, this is the ingraining of becoming part of the culture that we [want] to happen,” a Trainer shared (Trainer 6, Year 2).

## 4 Discussion

This study describes the development and implementation of a TIC initiative across health centers in Texas. Using a train-the-trainer model, TIC was disseminated throughout each participating health center, beginning with a year-long hands-on training of select Trainers that then shared their knowledge to Champions and Champions' knowledge to remaining staff. Prior literature indicates that TIC training is most effective when it is engaging, interactive, and actionable (31). Moreover, cross-departmental collaboration, such as with the team of Trainers, can strengthen providers' holistic knowledge of patient care and improve patient safety and quality of care (32). The inquiry-based curriculum, which emphasizes applied learning and reflection, may promote cultural competency and resilience among healthcare workers (33). The deemphasis on knowledge-based assessments allowed for enhanced descriptive feedback, greater intrinsic learner motivation, and reduced learner anxiety, especially when learners engaged with content they perceived as challenging (34, 35).

Trauma operates across multiple socioecological levels, including individual, interpersonal, and structural domains (36). Accordingly, evaluation across Trainers, coordinators, and organizations is necessary to fully capture program impact. Trainers demonstrated strong TIC knowledge with the inquiry-based approach, with an average of 73.9% correct responses on the summative assessments. Prior literature suggests that TIC knowledge is associated with better patient engagement, reduced stigma, and stronger patient-provider relationships (31, 37–39). Qualitative findings, however, indicate that implementation may still be limited by unequal staff engagement, insufficient leadership buy-in, and reduced confidence in training others (40).

Trainers and Champions provided valuable perspectives on TIC implementation. Although staff reported actively incorporating TIC principles into practice, Trainers had low self-efficacy in implementation. This disconnect likely reflects the abstract nature of TIC concepts and the challenges of translating them into routine clinical workflows (41). In contrast, staff wellness and self-care emerged as highly actionable components of the program, despite not being part of the original SAMHSA framework (23). These elements were well-received and may represent key drivers of engagement and cultural change. Trainers also appreciated the quick and thorough feedback they were given, an important aspect of effectively teaching clinicians (41). As a result of Trainer and Champion feedback, the curriculum was realigned for Cohort 4 and after to include cultural sensitivity and equity principles as well as remove content irrelevant to most staff (e.g., finance). Moreover, e-modules were piloted in select health centers in Cohorts 2 and 4 to accommodate issues with time and self-efficacy in training. Prior studies support the effectiveness of virtual TIC training in improving knowledge and confidence in similar settings (42–44). Sustainability of TIC implementation will depend on addressing key structural barriers, including time constraints, funding limitations, and leadership engagement. Greater involvement of organizational leadership, such as participation in training and active support of implementation, may improve resource allocation and facilitate integration into clinical workflows (45). Formalizing TIC training through certification or continuing education may further enhance participation and long-term adoption (46, 47).

The OA survey illustrated organizational and cultural changes in the health centers as TIC was implemented, and there were statistically significant improvements across most domains. Previous research shows the impact of TIC across all OA domains (48). Despite both the Trainers' and coordinators' suggestion that there was reduced self-efficacy in implementing TIC by Trainers and Champions, organizational transformation still occurred (40). Indeed, research shows that training that encompasses all levels of staff can produce organizational change and sustainability (49). The train-the-trainer model is additionally a proven method to produce long-term organizational change (50).

It is important to consider several limitations of this paper. The TIC initiative did not conduct a pretest-posttest design to assess significant differences in knowledge following the TIC Trainer training. We therefore cannot definitively conclude the knowledge gained was from the training, especially for those Trainers from a behavioral health background that may have previously been exposed to TIC principles. Health centers self-selected to participate in TIC, which may result in volunteer bias in which the health centers were already amenable to adopting TIC practices. Additionally, the train-the-trainer model assumes Trainers are sufficiently training Champions, and that Champions are adequately training the remaining staff; however, evaluation of staff-level trainings was not conducted due to the project's limitations in time and funding as well as to reduce health center staff burden to gather data. Nonetheless, previous research indicates the effectiveness of the train-the-trainer model in the healthcare setting, especially when it blends multiple learning approaches such as lectures, practical scenarios, and supporting materials (51). The TIC initiative implementation ended before Cohort 6 could participate in the OA and summative assessment; therefore, we do not have descriptive data on this Cohort's TIC experience. Furthermore, patient-level outcomes were not collected due to privacy, time, and funding limitations. Still, TIC uses a relationship-focused approach that has been shown to improve patient engagement and outcomes while reducing stigma in previous literature (52–54). Finally, this initiative was conducted in the FQHC setting and may not be generalizable to other health systems.

Future TIC programs should further tailor implementation to specific clinical settings and apply strategies to strengthen engagement with patients, staff, and leadership. Incorporating staff wellness and self-care remains a key strength of this program and should be maintained in future models. Additional research is needed to evaluate patient-level outcomes, including patient experience, satisfaction, and clinical outcomes associated with trauma-informed practices.

### 4.1 Conclusion

TIC has the potential to transform healthcare delivery to ensure staff, patient, and organizational wellbeing. Training healthcare organizations to provide care that considers past trauma and seeks to prevent retraumatization or vicarious trauma requires a multidisciplinary, socioecological approach. The development and implementation of this TIC initiative used a four-pronged curriculum to train a group of Trainers across FQHCs in Texas to implement TIC using a train-the-trainer model. With diverse FQHC participation, survey assessments showed improvements in organizational culture and Trainer knowledge upon completion of the TIC training. Qualitative findings highlighted both the successes and challenges of TIC implementation, including the successful integration of trauma-informed practices in healthcare delivery and staff wellness but also difficulties with leadership buy-in, funding, learner engagement, and time allocation.

## Data Availability

The datasets presented in this article are not readily available because the datasets presented in this article were from the primary care association “Texas Association of Community Health Centers”. Requests to access the datasets should be directed to Paula Cuccaro, paula.m.cuccaro@uth.tmc.edu.
